# Congenital cerebral malaria: a masquerader in a neonate

**DOI:** 10.1186/s12936-022-04056-2

**Published:** 2022-02-05

**Authors:** Ezinne I. Nwaneli, Chisom A. Nri-ezedi, Kenneth N. Okeke, Emeka S. Edokwe, Sylvia T. Echendu, Kenechukwu K. Iloh

**Affiliations:** 1 Department of Paediatrics, Faculty of Medicine, Nnamdi Azikiwe University, Awka, Anambra State Nigeria; 2 Department of Paediatrics, Nnamdi Azikiwe University Teaching Hospital, Nnewi, Anambra State Nigeria; 3 Life International Hospital, Awka, Anambra State Nigeria; 4 Department of Paediatrics, University of Nigeria Teaching Hospital, Ituku-Ozalla, Enugu State Nigeria

**Keywords:** Complicated malaria, Newborn, Seizures, Early onset neonatal sepsis, Neonatal meningitis

## Abstract

**Background:**

Congenital malaria, which is caused by vertical transmission of malaria parasites, is a potentially fatal condition. Despite Africa’s high malaria burden, congenital malaria is not routinely screened for, and thus may go undiagnosed. Malaria, if not treated promptly, can quickly progress to severe forms and result in death. Severe congenital malaria is believed to be uncommon in neonates due to maternal antibodies, fetal haemoglobin, and the placenta’s sieving effect. The majority of reported cases were classified as having severe anaemia. Following a thorough review of the literature, only one case of congenital cerebral malaria (CCM) has been reported, and it was misdiagnosed.

**Case presentation:**

A 5-day-old Nigerian neonate born to an apparently healthy mother initially displayed characteristics consistent with neonatal sepsis and severe neonatal hyperbilirubinaemia. He quickly developed characteristics consistent with meningitis. Surprisingly, the peripheral blood film revealed evidence of malaria parasites, which was immediately confirmed by Giemsa-stained thick and thin blood film microscopy for malaria. The patient was diagnosed with congenital cerebral malaria. The medication was modified to parenteral artesunate followed by oral artemisinin combination therapy. The neonate recovered fully and had no neurological deficits on follow up.

**Conclusion:**

Because CCM and infant meningitis have similar clinical presentations, CCM could be misdiagnosed and lead to death if there isn’t a high index of suspicion.

## Background

Malaria is primarily transmitted to humans through the bites of infected female Anopheles mosquitoes, but it can also be transmitted via blood transfusion, organ donation or from a pregnant woman to her fetus [1–3]. Malaria is acquired through mosquito bites following delivery [4]. Africa continues to have the world’s highest malaria burden and malaria is a significant public health problem in Nigeria, where it causes more cases and deaths than any other disease [5]. Available hospital review shows that malaria accounted for 60% of outpatient visits to health facilities, 30% of child mortality, 25% of infant mortality, and 11% of maternal mortality [6]. Repeated malaria infection results in the acquisition of agglutinating antibodies directed against the parasite-encoded variant surface antigen (VSA) expressed on parasitized red blood cells [7]. Repeated infection occurs in stable transmission areas, and thus residence in such areas provides some protection to the population.

In endemic areas such as Nigeria, where mothers have developed significant immunity to malaria, *Plasmodium falciparum* infection during pregnancy does not always result in symptomatic illness [8]. As a result, congenital malaria in an ill neonate may go unnoticed if the mother appears to be otherwise healthy. It is widely believed that the placenta serves as an effective barrier against malaria parasite transmission [9, 10]. Additionally, the newborn receives some immunoglobin G (IgG) antibodies against VSA from the mother, which provides some protection [7]. Furthermore, it has been demonstrated that fetal haemoglobin (HbF) protects against high parasitaemia, thereby preventing severe disease [11]. These features may contribute to physicians’ misdiagnosis of congenital and neonatal malaria, even in severe forms. It is worth noting, however, that estimated prevalence of congenital malaria was reported as 40.4/1000 in sub-Saharan Africa, based on a comprehensive review and meta-analysis of 22 observational studies by Dawang et al. [12]. This study complements a recent meta-analysis that examined 8148 newborns and estimated a global prevalence of 6.9% with considerable inter-country variability (with estimates as high as 46.7% in Nigeria) [13]. Furthermore, even in the absence of congenital malaria, placental malaria increases the risk of perinatal morbidity and mortality, including low birth weight, intrauterine growth restriction, premature labour, and intrauterine fetal death [14].

Congenital malaria is diagnosed by the detection of asexual malaria parasites on a blood smear of the newborn’s peripheral blood within the first 7 days of life [1, 15]. Passive immunity transmitted to the newborn may delay or alter the severity of symptoms for up to 6 weeks following delivery, making it difficult to differentiate between congenital and neonatal malaria [16]. Regardless of the clinical picture, neonates born to women who had malaria within the 7 days preceding birth should have a blood film checked for malaria parasites and be monitored weekly for the 1st month to increase the likelihood of early detection of congenital malaria [4].

Cerebral malaria is defined by the World Health Organization (WHO) as a clinical state characterized by a coma lasting at least 1 h after the end of a seizure or correction of hypoglycaemia, asexual *P. falciparum* parasites on peripheral blood smears, and no other explanation for the coma [17]. Physical signs of seizure activity in neonates are frequently imperceptible and subtle [18]. Subtle seizures account for half of neonatal seizures and are often difficult to distinguish from normal inter-ictal behaviors or physiological phenomena [19]. These include tonic, horizontal deviation of the eyes with or without jerking, blinking or fluttering of the eyelids, sucking, smacking, or other oral–buccal–lingual movements, swimming or pedaling movements, and, on rare occasions, apnoeic spells [20]. Failure to recognize the condition can be fatal, as these patients are hypoxic and hypercarbic due to hypoventilation and are at risk of aspiration [18]. If left untreated, cerebral malaria is always fatal [18]. Malaria during pregnancy is thought to be responsible for 100,000 neonatal deaths each year [8]. It is preventable during pregnancy with intermittent preventive treatment of malaria in pregnancy (IPTp) with sulfadoxine–pyrimethamine (SP), which has been shown to reduce neonatal mortality by up to 61% [21]. IPTp recommends a minimum of three SP doses, 1 month apart, starting after quickening (approximately 18 weeks gestation), and routinely delivered at antenatal clinics [22]. Nigeria adopted the IPTp strategy in 2005, with varying levels of implementation across the country’s states [23]. According to the 2018 Nigeria Demographic and Health Survey, 63% of women received at least one dose of SP, but 16.6% received at least three doses [24].

In endemic locales, the reported incidence of congenital malaria ranged from 0 to 37% [25–27], and majority of these cases were not serious and were discovered during routine neonatal screening. Severe anaemia is the most usually reported complication of malaria [25, 26, 28], Following a thorough examination of the literature, the investigators discovered only one incidence of congenital cerebral malaria, which occurred in India [29].

## Case report

A 5-day-old male neonate presented with 3 days history of yellowness of the body and 2 days history of poor suckling and excessive sleep. The yellowness of the body began on the face on the 2nd day of life and spread to other parts of the body. He sucked for less than 2 min on the mother’s breast on the 3rd day of life, compared to approximately 20 min prior to the illness. He was constantly asleep, and even when a perceived painful stimulus was applied, he cried poorly and low pitched. No fever or obvious convulsions were observed by the parents. Since birth, his mother had been administering 2.5 mls of oral paracetamol three times daily on the advice of a nurse. While pregnant, the mother received two doses of tetanus toxoid, but did not receive intermittent preventive treatment for malaria. She had no febrile illness throughout the pregnancy, but experienced foul-smelling creamy and itchy vaginal discharge in the final trimester, which was resolved with self-prescribed nystatin pessary. There was no prior history of premature membrane rupture. The pregnancy was carried to term, and the birth occurred vaginally. He was born crying and weighing 3.2 kg. The mother had no history of postpartum fever, and her lochia was not offensive. Due to a delay in lactation, the baby was given pre-lacteal feeds (infant formula) only on the 1st day of life, but by the second day, the baby was exclusively on breast milk and sucking effectively. On his 2nd day of life, he received BCG, oral polio 1, and hepatitis B virus vaccinations. He is the second child of civil servant parents, and his female elder sibling had no history of neonatal jaundice. The parents had O Rhesus positive blood groups, and there was no family history of glucose-6-phosphate dehydrogenase deficiency or the use of naphthalene balls on the baby’s clothing.

He was extremely irritable, in respiratory distress, febrile with an axillary temperature of 38 °C, moderately pale, icteric to the palms and soles, and acyanosed upon presentation. The respiratory rate (RR) was 68 breaths per minute, the heart rate (HR) was 162 beats per minute, the blood pressure (BP) was 102/51 mmHg, the oxygen saturation (SPO_2_) was 92 percent, and he weighed 2.98 kg. He had a Blantyre coma score (BCS) of 4/5 and a flat and soft anterior fontanelle (2.5 cm in diameter). He had normotonia but suboptimal primitive reflexes (Moro and palmar grasp). He also had hepatosplenomegaly and a wet but clean umbilical stump. The examination of the respiratory and cardiovascular systems revealed no abnormalities. A presumptive diagnosis of early onset neonatal sepsis and severe neonatal hyperbilirubinaemia was made. The laboratory result that was immediately available (random blood glucose) was 70 mg/dl. Serum bilirubin, urinalysis, urine microscopy, culture and sensitivity (m/c/s), umbilical swab m/c/s, complete blood count, peripheral blood film, direct and indirect Coombs tests, glucose-6-phosphate dehydrogenase, cerebrospinal fluid m/c/s, protein and glucose, serum electrolytes, urea and creatinine were also requested. He was started on intravenous ceftazidime (100 mg/kg) and geniticin (5 mg/kg), both in two divided doses 12 h apart, and a maintenance dose of 10% dextrose water. The patient received a double exchange blood transfusion (EBT) that was well tolerated. Following that, intensive phototherapy was continued. Breastmilk was fed 4 h after EBT via a nasogastric tube and was tolerated.

Thirteen hours into the admission, while still being monitored continuously for vital signs, his SPO_2_ was observed to decrease intermittently to approximately 30% to 40%, before increasing to approximately 80 to 90% after approximately 30 to 60 s. In 1 h, eight episodes were recorded. Further examination revealed that he was experiencing subtle seizures (apneic attacks) during periods when his SPO_2_ decreased. His RR was 64 to 70 breaths per minute (in between apneic attacks) and his HR was 120 beats per minute during the attack and increasing to 182 beats per minute afterwards. There was also an increase in his temperature (38.2 °C) and blood pressure (115/75 mmHg). His BCS was 2/5, his anterior fontanelle was tense, and he was hypertonic with an opthostonic posture. RBG was 118 mg/dl on repeat. The baby was placed on intranasal oxygen, and his drug prescription was modified to include three doses of intravenous (IV) mannitol (1 g/kg/dose over 30 min) 8 h apart, frusemide (2 mg/kg/dose to follow each dose of mannitol), diazepam (0.2 mg/kg/dose over 10 min), phenobarbitone (loading dose of 20 mg/kg, maintenance dose of 5 mg/kg in two divided doses 12 h apart), and dexamethasone 0.2 mg/kg/dose every 12 h. Even though apneic attacks did not recur following anticonvulsants, the patient maintained an opithostonic posture.

The peripheral blood film result (requested prior to the EBT) revealed normocytic, normochromic red blood cells, but also significant malaria parasites. A Giemsa-stained thick and thin blood film was immediately requested for malaria microscopy and revealed trophozoites of *P. falciparum* with a parasite density of 8341 parasites/µl. Other investigations conducted prior to the EBT revealed a total serum bilirubin of 30 mg/dl and conjugated bilirubin of 1.9 mg/dl. Complete blood count showed leucopenia of 2.0 × 10^9^/l, with differential neutrophils, lymphocytes and monocytes percentage of 30%, 69% and 1% respectively. Haematocrit level was 35.1% and he had thrombocytopenia (90,000/mm^3^). Urinalysis result revealed a deep amber and clear urine with presence of protein, urobilinogen and bilirubin. CSF analysis, urine m/c/s, umbilical swab m/c/s, serum electrolytes, urea and creatinine, both direct and indirect Coombs test returned no significant abnormality. Total serum bilirubin was 8.5 mg/dl following EBT, while conjugated bilirubin was 1.5 mg/dl. The patient was diagnosed with severe congenital malaria (cerebral malaria). Intravenous dexamethasone and antibiotics were stopped. Intravenous artesunate was prescribed at a dose of 3 mg/kg to be given at 0 h, 12 h and subsequently 24 hourly till the patient regains consciousness.

Following commencement of intravenous artesunate (Rekmal^®^), the patient’s consciousness gradually improved; by the second dose, he was no longer in an opisthotonic state, and by the fourth dose, he had regained complete consciousness and was able to suckle on his mother’s breast. After the fifth IV dose of artesunate, a 3-day course of oral artesunate–amodiaquine was initiated (artesunate, Artesunat^®^ 4 mg/kg/day and amodiaquine, Camoquin^®^ 10 mg base/kg/day). Intravenous phenobarbitone was gradually tapered off. The mother complained of a headache, fever, and chills on the 3rd day following presentation. A rapid diagnostic test for malaria histidine-rich protein 2 (HRP2) was positive, and she received a full course of ACT with clinical recovery. On the 5th day, the patient was discharged, and a repeat malaria microscopy performed that day was negative. The mother was counseled on the importance of intermittent preventive treatment during pregnancy, in addition to other malaria prevention measures. The patient was followed up for 1 year and no neurologic deficit was discovered during follow-up examinations of the central nervous system.

## Discussion

Congenital malaria is defined as the presence of malarial parasites in a baby’s cord blood or peripheral smear within 7 days of birth [4, 29]. This occurs as a result of vertical malaria parasite transmission prior to delivery. This case describes congenital malaria with symptoms beginning on the 3rd day of life and demonstrable hyperparasitaemia on the 5th day of life. Congenital malaria is reported to be uncommon, with the majority of cases occurring without symptoms [15, 26, 30] and approximately 7% to 10% of cases symptomatic [10, 31]. Congenital malaria has been documented to present with anaemia, fever, hepatosplenomegaly, poor feeding, lethargy, irritability, and jauncice [4, 16, 32, 33]. All the aforementioned symptoms were observed in the neonate who was also experiencing subtle seizures (apneic attacks), an unarousable coma, and opithostonic posturing. Following careful literature search, only a case of congenital cerebral malaria has been reported, and this was reported in Mumbai, India [29]. The Indian neonate presented on the 4th day of life with a history of fever and reduced feeding. Additionally, he was found to be irritable, febrile, icteric, exhibiting opisthotonic posture, tonic movements, hypertonic, and possessing a risus sardonicus-like facies [29]. The index neonate, like the Indian neonate, had hypertonia, and opithostonic posturing; however, the subtle pattern of their seizures was different. The neonate also had an unarousable coma which lasted more than 1 h.

Seizures and unarousable coma are known features of severe *P. falciparum* malaria referred to as cerebral malaria [18]. For cerebral malaria to be entertained in children, prolonged unconsciousness lasting more than an hour is required [18]. Due to the overlap of cerebral malaria and meningitis symptoms, a low index of suspicion, and a lack of screening, congenital malaria may be misdiagnosed as neonatal sepsis or neonatal meningitis, as was the case in the Nigerian and Indian neonates. In both cases, it was an unintentional examination of the peripheral blood smear to determine the red cell morphology of the icteric neonates that revealed haemoparasites within the red blood cells. Punta et al. reported a similar pattern of accidental discovery of malaria parasites in the blood of a neonate who was initially diagnosed with neonatal sepsis [34]. As required, the index patient was also examined for additional causes of altered consciousness, including hypoglycemia, meningitis, electrolyte imbalance, and uraemia [18], but none were found.

Malaria was confirmed using the gold standard of Giemsa-stained thick and thin blood film microscopy [35]. Although parasitaemia in newborns is typically low [15, 36, 37], this was not the case in the neonate who had high parasitaemia of 8341 parasites/µl [38, 39]. Vedang et al. also reported high parasitaemia with a parasite index of 5% in the Indian neonate [20]. The presentation of clinical disease in this Nigerian neonate by the 3rd day suggests that the transmission to the fetus may have occurred in utero even when the mother was asymptomatic. Relatively high in utero parasitaemia developed despite presumed maternal immunity and the reduced parasite growth rates associated with fetal haemoglobin [11]. The patient had full clinical and parasitological recovery following treatment with intravenous artesunate according to the WHO treatment guideline for complicated malaria [40]. The presence of maternal HRP2 antigenaemia confirmed maternal malaria infection. This confirms a previous finding that despite being asymptomatic, a mother living in a malaria-endemic area can transmit the parasite to her fetus. Given the speed with which malaria rapid antigen test results are available and the lack of specialized skill required for its performance when compared to malaria microscopy, it could also be used to screen sick neonates for malaria in the interim, while waiting for malaria microscopy, which requires expertise but is the gold standard for diagnosis. This will help in the early detection and treatment of congenital malaria. Although no guidelines exist to assess impairments or guide rehabilitation following cerebral malaria [41], a follow-up evaluation of the central nervous system of the patient was unremarkable.

## Conclusions

Cerebral malaria can present as a manifestation of congenital malaria and should be considered in patients who present with seizures and an unarousable coma. Sick neonates born to asymptomatic mothers in malaria-endemic areas should also be promptly investigated for malaria. Although additional data are needed in neonates, intravenous artesunate followed by oral artesunate–amodiaquine treatment appeared to be safe and effective.

## Data Availability

The clinical data of the patient is available upon request.

## Abbreviations

CCM: Congenital cerebral malaria
VSA: Variant surface antigen
IgG: Immunoglobin G
HbF: Fetal haemoglobin
IPTp: Intermittent presumptive treatment of malaria in pregnancy
BP: Blood pressure
SPO_2_: Oxygen saturation
BCS: Blantyre coma score
M/C/S: Microscopy, culture and sensitivity
HRP2: Histidine-rich protein 2
